# Thermodynamic vs. Kinetic Control in Synthesis of *O*-Donor 2,5-Substituted Furan and 3,5-Substituted Pyrazole from Heteropropargyl Precursor

**DOI:** 10.3390/molecules27165178

**Published:** 2022-08-14

**Authors:** Anton A. Muravev, Alexander S. Ovsyannikov, Gennady V. Konorov, Daut R. Islamov, Konstantin S. Usachev, Alexander S. Novikov, Svetlana E. Solovieva, Igor S. Antipin

**Affiliations:** 1 Infochemistry Scientific Center, ITMO University, 191002 St.-Petersburg, Russia; 2 Arbuzov Institute of Organic and Physical Chemistry, FRC Kazan Scientific Center, Russian Academy of Sciences, 420008 Kazan, Russia; 3 Butlerov Institute of Chemistry, Kazan Federal University, 420008 Kazan, Russia; 4 Institute of Fundamental Medicine and Biology, Kazan Federal University, 420008 Kazan, Russia; 5 Institute of Chemistry, St.-Petersburg State University, 199034 St.-Petersburg, Russia; 6 Joint Research Institute of Chemistry, Faculty of Physics, Mathematics and Natural Sciences, Peoples’ Friendship University of Russia (RUDN University), 117198 Moscow, Russia

**Keywords:** donor-substituted pyrazoles, 2,5-substituted furans, Sonogashira cross-coupling, tandem Michael addition/cyclocondensation, prototropic tautomerism, DFT calculations

## Abstract

Elaboration of a convenient route towards donor-substituted pyrazoles from heteropropargyl precursors is challenging due to a number of thermodynamically favorable side reactions (e.g., acetylene–allene isomerization and Glaser homocoupling). In this work, Sonogashira cross-coupling conditions of 4-*tert*-butylphenyl propargyl ether with benzoyl chloride followed by tandem Michael addition/cyclocondensation with hydrazine into 3,5-disubstituted pyrazole (kinetic control), as well as cycloisomerization conditions of ketoacetylene intermediate into 2,5-disubstituted furan (thermodynamic control), were established through a variation of the catalyst loading, solvent polarity, excess of triethylamine, and time of reaction. During the optimization of process parameters, a number of by-products represented by a monophosphine binuclear complex (PPh_3_PdI_2_)_2_ with two bridging iodine atoms and diyne were identified and isolated in the pure form. The quantum-chemical calculations and solution-state ^1^H/^13^C NMR spectroscopy suggested that the 5(3)-(4-*tert*-butylphenyloxy)methoxy-3(5)-phenyl-1*H*-pyrazole exists in the tautomeric equilibrium in a polar methanol solvent and that individual tautomers could be characterized in case aprotic solvents employed. The pyrazole features a unique tetramer motif in the crystal phase formed by alternating 3(5)-phenyl-1*H*-pyrazole tautomers, which was stabilized by N–H···N bonds and stacking interactions of pyrazole rings, whereas pyrazole dimers were identified in the gas phase.

## 1. Introduction

**Figure 1:**
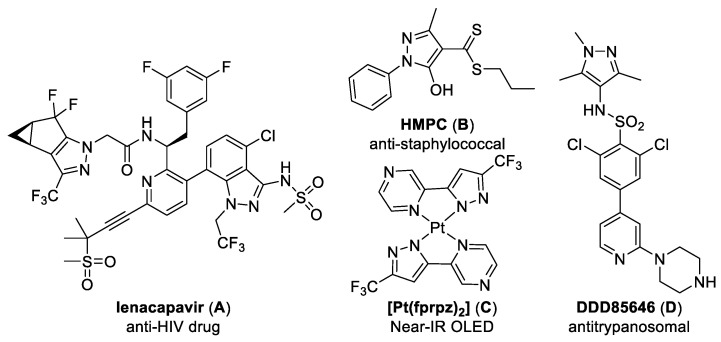
Some examples of biologically active and metal-coordinating pyrazoles.

1*H*-pyrazole is a promising scaffold for the design of compounds with anti-HIV [1], antimicrobial [2,3], and antifungal activity [4]. Of particular interest for medicinal chemistry are complex molecular structures with pyrazolyl moiety accessed via multi-component reactions [5]. Pyrazoles also feature coordinating ability towards ions and biomolecules [6,7], in particular those containing heteroatoms at C-3 or C-5 positions of heterocycles due to cooperative binding (compounds **A**–**D**) (Figure 1) [8,9,10].

**Scheme 1:**
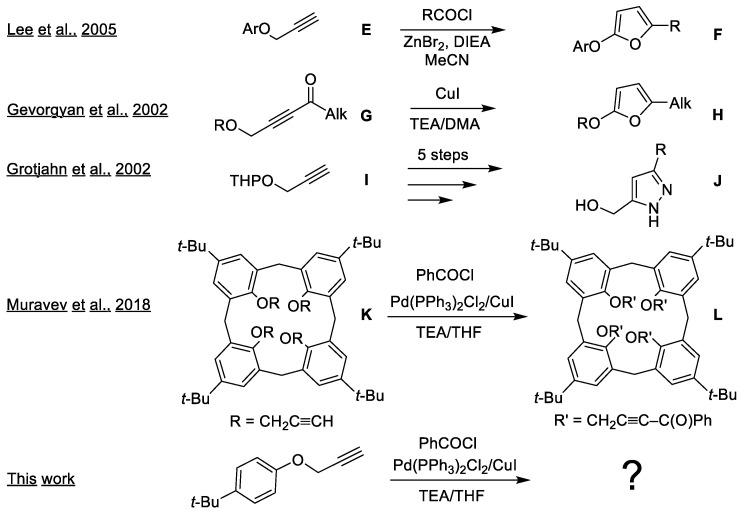
Synthetic routes towards furans [18,19] and pyrazoles [21,22] from heteropropargyl derivatives.

3,5-disubstituted 1*H*-pyrazole derivatives can be synthesized from ketoacetylenes and hydrazines, and a number of highly regioselective protocols involving metal catalyst or appropriate solvent were developed [11,12,13,14]. Ynone-based multicomponent reactions towards such pyrazoles, firstly reported by Mori’s group [15] and recently reviewed by Müller et al. [16], should also be noted. However, ketoacetylenes possessing a nearby methylene unit, generally with heteroatoms at the propargyl group (heteropropargyl systems), readily cycloisomerize into 2,5-disubstituted furans via acetylene–allene rearrangement (reactions **E**→**F** and **G**→**H**) (Scheme 1) [17,18,19,20]. Moreover, alternative approaches to their synthesis require expensive reagents or unstable precursors (aldehydes and diazo-compounds) or multiple-step synthesis involving protecting and deprotecting groups (reaction **I**→**J**) [21], which drastically reduces the accessibility of donor-substituted pyrazoles. Interestingly, in spite of facile cycloisomerization of ynones into furans in the presence of palladium or copper salts [19], no furans were detected during the Sonogashira reaction of aryl propargyl ethers on a 4-*tert*-butylcalix[4]arene scaffold with acid chlorides using 4 mol % Pd(PPh_3_)_2_Cl_2_/8 mol % CuI catalyst in a triethylamine (TEA)/THF solvent (reaction **K**→**L**) [22], presumably due to the steric hindrance of the macrocyclic platform. Thus, there is no clear statement on the result of Pd(II)–Cu(I)-catalyzed reaction of heteropropargyl systems with acid chlorides.

To the best of our knowledge, there are no works regarding the Sonogashira reaction conditions that favor the kinetic stability of conjugated ynones with electron-donating heteroatoms attached to the α-carbon of the triple bond. The aim of this work was to control the reactivity of heteropropargyl precursors towards the synthesis of electron-donating 2,5-substituted furans (thermodynamic control) or 3,5-disubstituted pyrazoles (kinetic control) via ketoacetylene intermediates using the propargyl ether of 4-*tert*-butylphenol as a model substrate. The optimization of the synthesis of 5-((4-(*tert*-butyl)phenoxy)methyl)-3-phenylpyrazole is particularly important, because it represents a model analog of the pyrazolyl derivatives of 4-*tert*-butylcalix[4]arene, which have previously displayed antitumor activity [23].

## 2. Results and Discussion

### 2.1. Optimization of Pyrazole and Furan Synthesis from the Heteropropargyl Precursor

**Scheme 2:**
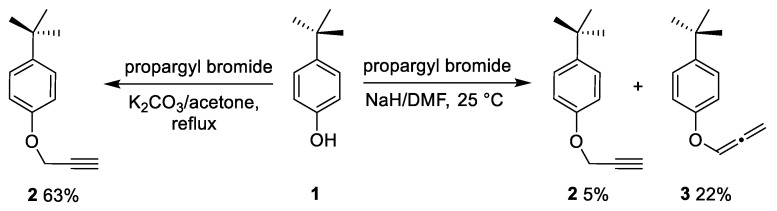
Synthesis of propargyl ether **2** and allenyl ether **3** of 4-*tert*-butylphenol.

The alkylation of 4-*tert*-butylphenol **1** with propargyl bromide in a NaH/DMF system [24] gave ether **2** only at a 5% yield (lit. 68%), while the major product of reaction was allene **3** (yield: 22%), the product of acetylene–allene isomerization driven by proton migration in a superbasic medium of NaH/DMF (Scheme 2). The allenyl group was clearly identified in NMR spectra due to resonances of CH_2_ and CH protons at 5.46 and 6.86 ppm, respectively, and the ^13^C NMR signal of the *ipso*-C atom at 203.0 ppm (Figures S1 and S2). The product was also identified by the mass peak at *m*/*z* 206.0 corresponding to the [M + NH_4_]^+^ ion (Figure S3). The same reaction under milder conditions (K_2_CO_3_) [25] afforded only propargyl ether **2** at a 63% yield.

**Scheme 3:**
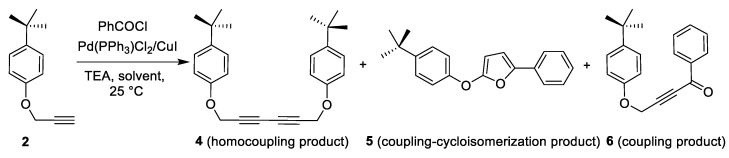
Outcome of reaction between aryl propargyl ether **2** and benzoyl chloride.

The Sonogashira reaction of aryl propargyl ethers with acid chlorides using the Pd(PPh_3_)_2_Cl_2_/CuI catalyst in the TEA/THF solvent was the starting point of the optimization of cross-coupling of ether **2** and PhC(O)Cl (Scheme 3). The thin-layer chromatography of the reaction mixture revealed a spot at *R*_f_ ≈ 0.6 with an eluent of a hexane:ethyl acetate at a 4:1 volume ratio. The slow evaporation of the corresponding fraction isolated after column chromatography produced X-ray quality crystals, the structure refinement of which gave the molecular formula of the di-*µ*-iodo-bridged dimer (PPh_3_PdI_2_)_2_, identical to that of the one previously isolated as a side product of cross-coupling reaction (Figure S4) [26]. Given that the products of transformation of aryl propargyl ether **2** (homocoupling into diyne **4**, cross-coupling followed by cycloisomerization into furan **5** via acetylene–allene rearrangement, and cross-coupling into ynone **6**) could be easily distinguished from each other and unreacted alkyne **2** by the splitting pattern of CH or CH_2_ protons in ^1^H NMR spectra, this technique was further employed to analyze the reaction outcome (Table 1; Figure S5). The effect of the [Pd]/[Cu] catalyst loading (in mol %) was firstly evaluated (entries 1–3). At a 1% [Pd]/2% [Cu] loading (corresponding to that in case of tetrapropargyl calixarenes [23] per one propargyl unit), the conversion of alkyne **2** was 6% after 12 h of stirring, and there was only the homocoupling product **4** represented by the singlet of CH_2_-protons at 4.73 ppm [27]. A full conversion of precursor **2** was achieved with an increase in the catalyst loading up to 2% [Pd]/4% [Cu]. In this case, the major product of reaction was represented by furan **5**, which was further isolated at a 52% yield (Figures S6–S8). Proton resonances of furan heterocycle appeared as an AB system (two doublets at 6.64 and 5.67 ppm). A subsequent increase in the catalyst loading to 4% [Pd]/8% [Cu] (entry 3) did not alter the reaction outcome.

The identification of furan **5**, instead of ynone **6**, in the reaction mixture indicated a low activation barrier of ynone-to-allenone isomerization in dipolar aprotic THF and suggests that nonpolar solvents could destabilize previously postulated charged carbanion transition state [19], leading to compound **5**. Indeed, the replacement of THF by less polar CH_2_Cl_2_, TEA, PhCH_3_, and hexane (entries 4–7) decreased the furan **5** content in the reaction mixture from 98% in THF to 14% in hexane, whereas the ynone **6** content gave a rising trend from 0% to 83%. The formation of ynone **6** was revealed by a singlet at 4.96 ppm corresponding to the protons of CH_2_ group; however, the ether group of ynone **6** was cleaved, and 4-*tert*-butylphenol **1** was formed at the purification stage using column chromatography. Reaction time is another optimization parameter that could raise the furan-to-ynone ratio also in nonpolar solvents. Indeed, an increase in the stirring time in hexane from 12 h to 72 h led to a notable increase in the furan content from 14% (entry 7) to 95% (entry 8), whereas no traces of furan **5** were detected at a shorter reaction time corresponding to a 64% conversion of alkyne **2** (3 h, entry 9). A similar trend was observed with a substitution of THF for hexane, showing a higher content of ynone **6** after 3 h (entries 2 vs. 10). Thus, the optimization data indicated that furan **5** is a thermodynamically controlled product whereas ynone **6** is a kinetically controlled product.

In contrast to that of furan **5**, the formation of diyne **4** could not be controlled by the catalyst loading, nature of solvent, and time of reaction; however, an increase in its content in the reaction mixture using TEA as a solvent (entry 5) suggested that a decrease in the amount of TEA can disfavor Glaser homocoupling. In fact, with only 1.2 eq. TEA in hexane, the ynone-to-diyne ratio was higher than 15, and no furan was detected in the reaction mixture (entry 11). Furthermore, a decrease in TEA amount eliminated the formation of furan **5** even in THF (entry 12), which indicated a significant role of the excess of base in ynone–furan cycloisomerization. In spite of this fact, THF increased the diyne content (~20%), likely due to the higher solubility of oxygen in it; this did not make THF the optimal solvent for the cross-coupling of propargyl ether **2**.

**Scheme 4:**
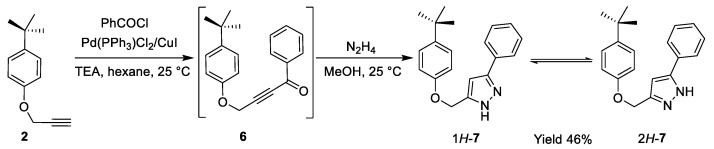
One-pot reaction of ether **2** with benzoyl chloride and hydrazine towards pyrazole **7**.

Given the low stability of ynone **6** at the purification stage, a one-pot reaction of alkyne **2** with PhCOCl under optimal conditions (Table 1, entry 11) over a longer period of 24 h (in order to achieve a higher conversion of alkyne **2**), followed by the addition of 6 eq. N_2_H_4_ in MeOH, was suggested and realized (Scheme 4). The ^1^H NMR spectrum of the reaction mixture (Figure S9) showed that the pyrazole **7**-to-furan **5**-to-diyne **4** ratio was 91:8:1 and no traces of starting alkyne **2** were detected. Signals of furan **5** presumably arose from the change of solvent polarity at the second stage. The column chromatography of the crude residue afforded pyrazole **7** at a 46% yield.

### 2.2. Structural Characterization of Pyrazole ***7***

**Figure 2:**
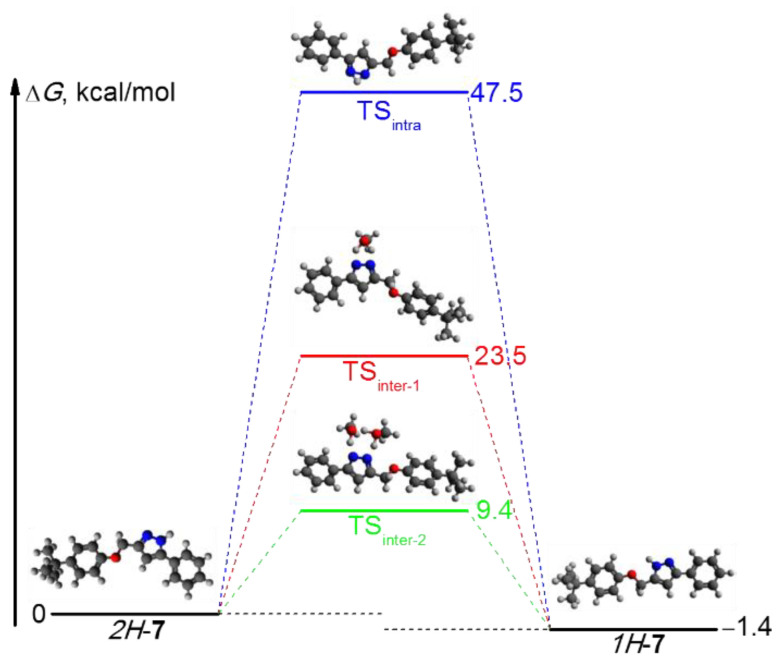
Calculated values of Gibbs free energies of activation (Δ*G*^≠^) and reaction (Δ*G*) for intramolecular and intermolecular (solvent-assisted) tautomerization of pyrazole **7**.

Analysis of the molecular and supramolecular structure of compound **7** could be difficult, because NH-pyrazoles undergo prototropic tautomerization as shown in Scheme 4 and could form different intermolecular H-bonding patterns, which are affected by many factors including solvents, phase states, and temperature. To evaluate the kinetic favorability of tautomerization from 1*H*-pyrazole into 2*H*-pyrazole, quantum-chemical calculations of the activation barrier were carried out (computational details are given in the Materials and Methods section and in Table S1, Supplementary Materials). Such tautomerism is possible in the form of both intramolecular proton migration and solvent-assisted proton migration, which requires an assumption of solvent molecules, such as methanol, which was present in the reaction mixture as a solvent. Computations showed the following results (Figure 2). Firstly, 1*H*-tautomer of compound **7** was thermodynamically more favorable than 2*H*-tautomer by 1.4 kcal/mol in terms of Gibbs free energy Δ*G*. The relationship between the difference of the total electronic energy of tautomers Δ*E* and the thermodynamic equilibrium constant *K_T_* via the Arrhenius equation provided a *K_T_* value of 3.87, which was the estimate of the tautomer ratio in the reaction mixture. Secondly, calculations indicated that the Gibbs free energy of activation for intramolecular 2*H*-**7** → 1*H*-**7** transformation (Δ*G*^≠^ = 47.5 kcal/mol) was much larger than that involving solvent molecules 2*H*-**7** + MeOH → 1*H*-**7** + MeOH (Δ*G*^≠^ = 23.5 kcal/mol). Moreover, the inclusion of another methanol molecule into the transition state lowered the Gibbs energy by up to 9.4 kcal/mol, which indicated the possibility of the tautomeric equilibrium in a MeOH solution at room temperature. Thus, considering the low activation barrier of solvent-assisted 2*H*-**7** → 1*H*-**7** tautomerism in methanol, one can expect the formation of both pyrazole tautomers; on the other hand, a sufficiently high activation barrier for intramolecular transition in the gas phase or in a hexane−ethyl acetate eluent or deuterated chloroform could rationalize the isolation of individual fractions of both tautomers after chromatography and the absence of tautomerization at an NMR time scale.

**Figure 3:**
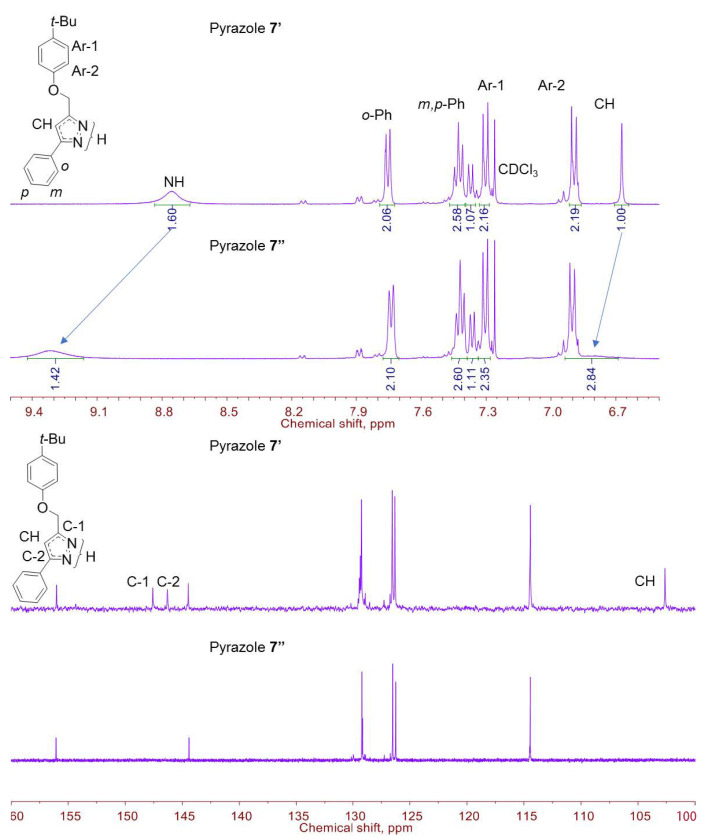
Fragments of ^1^H (6.5–9.5 ppm) and ^13^C NMR spectra (100–160 ppm) of pyrazole **7**.

The column chromatography of pyrazole **7** in a hexane–ethyl acetate eluent allowed us to isolate two fractions with *R*_f_ of 0.39 (fraction **7**′; 34% yield) and *R*_f_ of 0.42 (fraction **7**″; 12% yield). The IR spectra of both fractions recorded in a KBr pellet showed a broad band in the range of 3300–3000 cm^−1^, which indicated supramolecular interactions in the solid state through H-bonding (Figure S10). This suggestion was supported by the ESI mass spectrum of fraction **7**″ in the gas phase, which recorded a dimer ion peak with *m*/*z* 613.2 [2M + H]^+^ along with the molecular ion peak at *m*/*z* 306.6 [M + H]^+^ (Figure S11). The ^1^H NMR spectrum of fraction **7**′ was typical of 3,5-disubstituted 1*H*-pyrazoles and was characterized by the singlet of the CH-group of the pyrazolyl ring at 6.67 ppm, whereas NH-proton appeared as a broad signal at 8.76 ppm (Figure 3 and Figure S12). The ^13^C NMR spectrum of the product showed resonances of pyrazolyl rings at 147.6, 146.3, and 102.6 ppm, which confirmed the absence of the dynamic equilibrium between two pyrazole tautomers in a 0.05 M solution of this fraction in CDCl_3_ at 298 K (Figure 3 and Figure S13). Interestingly, resonances of ^1^H and ^13^C nuclei of fraction **7**″ coincided with those of fraction **7**′, except for the signals of pyrazolyl ring (Figure 3, Figures S14 and 15). More specifically, the ^1^H NMR spectrum showed broader resonances of NH and CH protons with their downfield shifts (by 0.56 and 0.15 ppm, respectively). In addition, the ^13^C NMR spectra of that fraction revealed a “selective” disappearance of the signals of pyrazolyl unit. Such a behavior was previously observed during the dynamic complexation of triazoles with impurity paramagnetic Cu^2+^ ions [28]. However, no peaks of metal complexes were recorded in the mass-spectrum of fraction **7**″ and X-ray fluorescence spectra did not indicate the presence of Cu and Pd atoms. The absence of NMR resonances of *ipso*-carbon nuclei of 2*H*-pyrazolyl unit was previously reported in [29]; however, the origin of such a phenomenon was not rationalized. Alternatively, the downfield shifts and broadening of the ^1^H NMR resonances of pyrazole ring suggested the formation of the H-bonded associate consisting of both 3-phenyl-1*H*-pyrazole and 3-phenyl-2*H*-pyrazole units in fraction **7**″, in which the tautomers did not interconvert due to the high tautomerization barrier in CDCl_3_.

**Figure 4:**
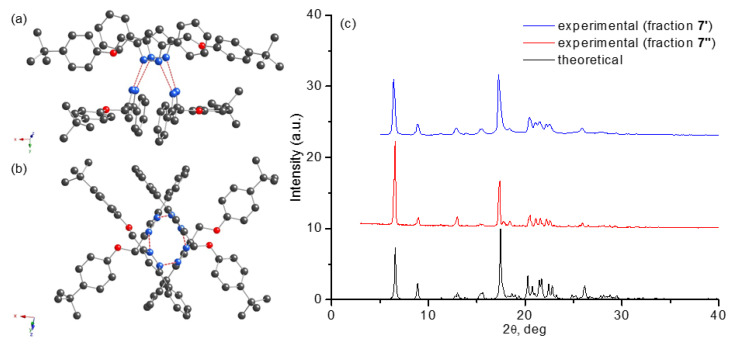
Side view (**a**) and top view (**b**) of hydrogen-bonded tetramer associates of compound **7** in crystal. Red dashed lines show H-bonds between N atoms of neighboring pyrazolyl units. C atoms are represented by gray spheres; N atoms are presented in blue; and O atoms are displayed in red. Disordered atoms of C, O, and H are not given. (**c**) Powder X-ray diffractograms of compound **7** (experimental and theoretical).

A further evidence of H-bond formation was gained from the refinement of the structure of pyrazole **7** isolated from fraction **7**″ as needle-like crystals using X-ray diffractometry. This compound crystallized in the triclinic space group *P*-1, with four independent conformers of compound **7** in the unit cell, which differed by torsion angles N–C–C–O (N13–C18–C62–O111 57.5(2)°, N53–C6–C42–O27 95.8(1)°, N51–C44–C68–O55 8.3(2)°, and N105–C36–C52–O109 90.8(1)°) (Figure 4a,b). Phenolic fragments and *tert*-butyl groups of two conformers out of four were disordered in the crystal. The planes of pyrazolyl and aryl fragments were nearly parallel in all conformers and dihedral angles between these planes are 2.95°, 4.04°, 1.23°, and 6.46°. The analysis of the secondary structure in the crystal revealed the formation of cyclic tetramer hydrogen-bonded associates, which are typical of pyrazoles with bulky substituents at 3- and 5-positions [30], due to the presence of the electron-withdrawing nitrogen atom and the electron-donating NH group in the pyrazole ring. In this associate, each pyrazole unit was linked via a H-bond to the N atom of the pyrazolyl ring of two neighboring molecules of compound **7**; in this case, the N–N distance corresponded to 2.895(1) Å, 2.822(1) Å, 2.942(2) Å, and 2.788(2) Å (Figure 4a). The formation of the intermolecular H-bond could observe a π-stacking interaction between pyrazolyl rings of the tautomer with the distances between C_3_N_2_-centroids of 3.507 Å and 3.764 Å (their dihedral angles were 16.96° and 8.99°, respectively). Each π-stacked dimer fragment was nearly perpendicular to another dimer (Figure 4b). The interaction of the tetramers along 0*x* axis resulted in the formation of a 1D chain via relatively weak intermolecular CH–π interactions between phenolic and pyrazolyl groups (3.831 Å and 4.097 Å). The disordering of the proton between two identical N atoms of neighboring molecules in the crystal presumably due to the solid-state proton transfer indicated the presence of both pyrazole **7** tautomers, which is a very rare case in the crystal structure of 3,5-substituted pyrazoles. While the alternating sequence of these tautomers (1*H*-2*H*-1*H*-2*H*) is a unique tetramer motif composition among the pyrazoles (previous examples include a 1*H*-1*H*-2*H*-2*H* sequence for 3-methyl-5-phenylpyrazole [31] and a 1*H*-1*H*-1*H*-2*H* sequence for 3-ethyl-5-phenylpyrazole [32]), this result discarded the hypothesis that fraction **7**″ in solutions is represented exclusively by the 2*H*-tautomer of compound **7**, according to which there is a transition from the 1*H*-tautomer to the energetically less favorable 2*H*-tautomer upon a dissolution of the 1*H*-2*H*-1*H*-2*H* tetramer in low-polar CDCl_3_.

The recorded powder X-ray diffractogram of fraction **7**″ (Figure 4c, red line) indicated a high degree of crystallinity of the specimen (91%), and it closely matched the one simulated from single-crystal X-ray diffractometry (black line), which indicated the presence of only one individual compound in the powder and supported the statement on the absence of paramagnetic metal impurities in the specimen (Figure 4c). Interestingly, the powder X-ray diffractogram of fraction **7**′ possessed nearly the same pattern (blue line) as that of fraction **7**″, which suggested that the 1*H*-tautomer of pyrazole **7** (compound **7**′) was also assembled into a H-bonded tetramer, but consisted only of 1*H*-tautomer units.

**Scheme 5:**
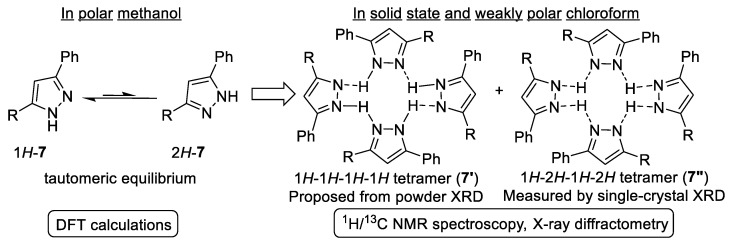
Proposed pyrazole **7** structures (R = 4-*t*-BuPhOCH_2_) under different conditions.

Thus, the combined theoretical, NMR spectroscopy, and X-ray crystallography study of pyrazole **7** indicated the formation of both tautomers during the kinetically controlled cross-coupling of propargyl aryl ether **2** and benzoyl chloride followed by condensation with hydrazine. IR spectroscopy, ESI mass spectrometry, and X-ray diffraction data revealed a supramolecular association of pyrazole **7** units through H-bonding. The tautomeric behaviors of pyrazole **7** under different conditions are summarized in Scheme 5.

## 3. Materials and Methods

Solvents were purified using known procedures [33]; the reagents were used as received. Ether **2** was synthesized following the literature procedure [25]. Physical constants of compounds **2** and **4** are given in the literature [25,27] and coincide with the data in this work. The structures of the compounds were elucidated using a set of physical methods. NMR spectra were recorded using Avance 600 (600.13 MHz for ^1^H, 150.90 MHz for ^13^C) at 303 K (compound **7**) and Avance 400 (399.93/400.13 MHz for ^1^H and 100.61/100.56 MHz for ^13^C) spectrometers of Bruker Company (Ettlingen, Germany) at 298 and 303 K (compounds **3** and **5**, respectively). As an internal standard, CDCl_3_ was used (δ_H_ 7.26 ppm; δ_C_ 77.16 ppm). ^1^H NMR spectra were accumulated using the zg30 pulse program (the acquisition time was 1.7 s, and the relaxation delay was 3.0 s). ^13^C NMR spectra were accumulated using the zgdc30 pulse program (the acquisition time was 0.7 s, and the relaxation delay was 3.0 s). Molecular masses (*m/z*) were detected with a Bruker Amazon X mass spectrometer (Bremen, Germany) in 0.1% NH_4_OAc in MeOH. IR spectra were recorded with a Bruker Vector-22 spectrometer (Ettlingen, Germany) in the pure form (allene **3**) or in a KBr pellet (compounds **5** and **7**). Elemental composition was determined with a Hekatech EA 3000 Euro Vector CHNSO-analyzer (Haaksbergen, Netherlands). Melting points were determined on a Boetius heating table (Dresden, Germany). Powder X-ray diffraction data of pyrazole **7** fractions were collected using a Bruker D2 Phaser diffractometer (Karlsruhe, Germany) with Cu*K*_α_ radiation (λ = 1.5406 Å).

1-(*Tert*-butyl)-4-(propa-1,2-dien-1-yloxy)benzene **3**: To a suspension of 4.32 g NaH (180 mmol) in 50 mL of DMF at 0 °C, a total of 9.01 g of 4-*tert*-butylphenol (60 mmol) were added portionwise over 30 min, and the reaction mixture was further stirred for 1 h under a nitrogen flow. Then, a total of 13.58 g of 80 wt % of a propargyl bromide solution in toluene (120 mmol) were added dropwise at vigorous stirring. After 24 h of stirring at 25 °C, the solvent was removed under vacuum, and the residue was extracted with diethyl ether. The organic phase was washed with water and brine and dried over Na_2_SO_4_. After the filtration of desiccant and the removal of solvent, the crude residue was purified by silica gel chromatography in a hexane–ethyl acetate eluent, yielding the title compound as a colorless oil (2.50 g, 22%). *R*_f_ (hexane:ethyl acetate = 100:1) was 0.54. ^1^H NMR (CDCl_3_): 7.35 (2H, m, H_Ar_), 7.02 (2H, m, H_Ar_), 6.86 (1H, td *J* 2.0, 6.0, CH), 5.46 (2H, dd *J* 1.6, 6.0, CH_2_), and 1.34 (9H, s, CH_3_). ^13^C NMR (CDCl_3_): 31.6, 34.4, 89.6, 116.3, 118.2, 126.4, 145.7, 155.1, 203.0. *m/z* (ESI) (%) 205 (100) [M − H + NH_4_]^+^. Anal. calcd. for C_13_H_16_O: % C 82.94, H 8.57; found: C 82.99, H 8.58. IR ($\tilde{\nu }$/cm^−1^) 2961 s, 1607 s, 1511 s, 1238 s, and 1180 s.

2-(4-(*Tert*-butyl)phenoxy)-5-phenylfuran **5**: To a pre-mixed suspension of 80 mg of CuI (4 mol %) and 150 mg of PdCl_2_(PPh_3_)_2_ (2 mol %) in 50 mL THF, a total of 2.00 g of propargyl ether **2** (10.62 mmol), 6.50 mL of triethylamine (46.64 mmol), and 1.64 g of PhC(O)Cl (11.69 mmol) were added. The orange suspension was stirred at 25 °C for 24 h. After the filtration of ammonium salt and the removal of the solvent from the filtrate, the crude oil was purified by silica gel chromatography in a hexane eluent, yielding the title compound as a white powder (1.61 g, 52%). *T*_m_ was 71 °C. *R*_f_ (hexane) was 0.33. ^1^H NMR (CDCl_3_): 7.64 (2H, m, H_Ar_), 7.39 (3H, m, H_Ar_), 7.26 (1H, m, H_Ar_), 7.10 (2H, m, H_Ar_), 6.64 (1H, d *J* 3.2, H_fur_), 5.67 (1H, d *J* 3.2, H_fur_), and 1.37 (9H, s, CH_3_). ^13^C NMR (CDCl_3_): 31.6, 34.5, 90.9, 106.3, 116.7, 123.1, 126.7, 127.0, 128.8, 130.8, 146.3, 147.0, 154.7, and 156.7. *m/z* (ESI) (%) 328 (100) [M − H + 2NH_4_]^+^. Anal. calcd. for C_20_H_20_O_2_: % C 82.16, H 6.90; found: C 82.38, H 6.59. IR (KBr, $\tilde{\nu }$/cm^−1^) 2965 s, 1548 s, 1506 s, 1245 s, and 1167 s.

5(3)-((4-(*Tert*-butyl)phenoxy)methyl)-3(5)-phenyl-1*H*-pyrazole **7**: The first stage of the process repeated that of the synthesis of furan **5** using hexane as a solvent and 1.61 g of TEA (15.93 mmol). To a crude residue, which was obtained after the removal of the solvent, a total of 2.01 g of N_2_H_4_ (62.73 mmol) and MeOH (30 mL) were added, and the reaction mixture was further stirred at 25 °C for 12 h. After the removal of the solvent under vacuum, the crude residue was purified using column chromatography in a hexane–ethyl acetate eluent, yielding the title compound as yellow crystals (1.50 g, 46%). *T*_m_ was 121 °C. *m/z* (ESI) (%) 307 (83) [M + H]^+^, and 613 (100) [2M + H]^+^. Anal. calcd. for C_20_H_22_N_2_O: % C 78.40, H 7.24, and N 9.14; found: C 78.55, H 7.22, and N 9.16. IR (KBr, $\tilde{\nu }$/cm^−1^) 3096 s, 2959 s, 1608 s, 1512 s, 1240 s, and 1187 s. *1H-tautomer (3-Ph)*. The yield was 34%, and *R*_f_ (*n*-C_6_H_14_:EtOAc = 2:1) was 0.39. ^1^H NMR (CDCl_3_): 8.76 (1H, br s, NH), 7.75 (2H, m, H_Ar_), 7.43 (2H, m, H_Ar_), 7.37 (1H, m, H_Ar_), 7.30 (2H, m, H_Ar_), 6.89 (2H, m, H_Ar_), 6.67 (1H, s, H_4_), 5.13 (2H, s, CH_2_), and 1.30 (9H, s, CH_3_). ^13^C NMR (CDCl_3_): 31.6, 34.3, 62.7, 102.6, 114.4, 126.3, 126.5, 129.2, 129.3, 129.4, 144.5, 146.3, 147.6, and 156.0. *1H-2H-1H-2H-tetramer*. The yield was 12%, and *R*_f_ (*n*-C_6_H_14_:EtOAc = 2:1) was 0.42. ^1^H NMR (CDCl_3_): 9.32 (1H, br s, NH), 7.75 (2H, m, H_Ar_), 7.43 (2H, m, H_Ar_), 7.37 (1H, m, H_Ar_), 7.30 (2H, m, H_Ar_), 6.89 (2H, m, H_Ar_), 6.82 (1H, br s, H_4_), 5.13 (2H, s, CH_2_), and 1.30 (9H, s, CH_3_). ^13^C NMR (CDCl_3_): 31.6, 34.3, 62.7, 114.4, 126.3, 126.5, 129.2, 129.3, 129.4, 144.5, and 156.0.

The data set for the single crystal of compound **7** was collected on a Rigaku XtaLab Synergy S instrument (Tokyo, Japan) with a HyPix detector and a PhotonJet microfocus X-ray tube using Cu*K*α radiation (1.54184 Å) at low temperature. Images were indexed and integrated using the CrysAlisPro data reduction package. Data were corrected for systematic errors and absorption using the ABSPACK module: numerical absorption correction based on Gaussian integration over a multifaceted crystal model and empirical absorption correction based on spherical harmonics according to the point group symmetry using equivalent reflections. The GRAL module was used for analysis of systematic absences and space-group determination. The structure was solved by direct methods using the SHELXT program [34] and refined by the full-matrix least-squares on F^2^ using the SHELXL program [35]. Non-hydrogen atoms were refined anisotropically. The hydrogen atoms were inserted at the calculated positions and refined as riding atoms. The figures were generated using Mercury 4.1 program [36]. The crystals were obtained by the slow evaporation method from a hexane–ethyl acetate solvent (*v*/*v*: 2:1).

Crystallographic data for C_20_H_22_N_2_O (*M* = 306.39 g/mol): triclinic, space group *P*-1 (no. 2), *a* = 11.73106(11) Å, *b* = 14.94073(16) Å, *c* = 20.7977(3) Å, *α* = 73.0170(10)°, *β* = 87.4630(9)°, *γ* = 78.4469(9)°, *V* = 3415.23(7) Å^3^, *Z* = 8, *T* = 100.0(3) K, *μ*(CuKα) = 0.577 mm^−1^, *D*_calc_ = 1.192 g/cm^3^, 106727 reflections measured (4.442° ≤ 2 *θ* ≤ 153.418°), 13707 unique (*R*_int_ = 0.0334, *R*_sigma_ = 0.0159) which were used in all calculations. The final *R*_1_ was 0.0455 (I > 2σ(I)), and *wR*_2_ was 0.1175 (all data). CCDC number: 2063297.

The full geometry optimization of all model structures was carried out at the M06-2X/6-31G* level of the theory with the help of the Gaussian-09 program package [37]. No symmetry restrictions were applied to the geometry optimization procedure. The Hessian matrices were calculated analytically for all optimized model structures to prove the location of the correct minimum or saddle point (transition state) on the potential energy surface (no imaginary frequencies or only one imaginary frequency, respectively). Chemcraft program [38] was used to visualize imaginary frequencies in transition states (see appropriate attached gif-files). The thermodynamic parameters were calculated at 298.15 K and 1.00 atm. Cartesian atomic coordinates for all optimized equilibrium model structures are presented in Supplementary Materials as xyz-files.

## 4. Conclusions

In this work, the conditions affecting the outcome of palladium-catalyzed reaction between propargyl ether of 4-*tert*-butylphenol and benzoyl chloride have been established through a variation of the catalyst loading, the nature of solvents, reaction time, and excess of a triethylamine base. To yield exclusively a thermodynamic product, 2,5-disubstituted furan, a long-term stirring in excess of triethylamine base (4 eq.) was sufficient, while the kinetic control, with the formation of ketoacetylene, could only be realized at a slight excess of triethylamine (1.2 eq.). In the case of kinetic control, nonpolar solvents, such as hexane, were desirable to avoid the Glaser homocoupling of starting alkyne. A one-pot procedure based on a kinetically controlled Sonogashira reaction towards ynone and the cyclocondensation of the ynone with hydrazine has been developed for the formation of 5-((4-(*tert*-butyl)phenoxy)methyl)-3-phenylpyrazole, which offered a convenient route to unsymmetrically 3,5-substituted NH-pyrazoles with electron-donating heteroatoms attached to the C3 or C5-position of the pyrazole ring via a methylene spacer. The X-ray diffractometry and ^1^H/^13^C NMR spectroscopy studies of the synthesized pyrazole, aided by quantum-chemical calculations, have revealed that there was a low energy difference between 1*H*- and 2*H*-tautomers, but a high activation barrier of their interconversion in nonpolar medium has allowed experimentally isolating the product in two desmotropic forms. The first desmotrope was represented by exclusively 1*H*-tautomer, whereas the second one was a H-bonded tetramer associate formed by two molecules of 1*H*-tautomer and two molecules of 2*H*-tautomers. The identical reflexes of both desmotropes in powder X-ray diffractograms allow us to suggest that the first desmotrope consisting of 1*H*-pyrazole also formed a tetrameric motif in the solid state.

## Figures and Tables

**Table 1:** Optimization of the cross-coupling of propargyl ether **2** with PhC(O)Cl.

Entry	Pd/Cu (mol %)	Solvent	t (h)	TEA (mol %)	Percentage of Unreacted Alkyne and Coupling Products in the Reaction Mixture ^1^			
					Alkyne 2	Diyne 4	Furan 5	Ynone 6
1	1%/2%	THF	12	440	94	6	–	–
2	2%/4%	THF	12	440	–	2	98	–
3	4%/8%	THF	12	440	–	3	97	–
4	2%/4%	CH_2_Cl_2_	12	440	–	5	85	10
5	2%/4%	TEA	12	5000	14	16	50	20
6	2%/4%	PhCH_3_	12	440	–	7	15	78
7	2%/4%	C_6_H_14_	12	440	–	3	14	83
8	2%/4%	C_6_H_14_	72	440	–	5	95	–
9	2%/4%	C_6_H_14_	3	440	36	8	–	56
10	2%/4%	THF	3	440	–	5	66	29
11	2%/4%	C_6_H_14_	12	120	51	3	–	46
12	2%/4%	THF	12	120	–	20	–	80

^1^ Proton resonances of phosphine ligands from catalyst species were not considered, because they could hardly be distinguished from the signals of aryl protons corresponding to the products of reaction. The component ratio in reaction mixture was calculated from the integral intensity of the peaks of methylene units of alkyne **2**, diyne **4**, and ynone **6** and those of the furan ring of compound **5**.

## Data Availability

The data presented in this study are available in the article and Supplementary Materials.

## Supplementary Materials

Supplementary Materials can be found at https://www.mdpi.com/article/10.3390/molecules27165178/s1. Figure S1: ^1^H NMR spectrum of compound **3** (CDCl_3_, 400 MHz, 298 K), Figure S2: ^13^C NMR spectrum and DEPT-135 experiment of compound **3** (CDCl_3_, 100 MHz, 303 K), Figure S3: ESI mass spectrum (0.1% NH_4_OAc, MeOH) of allene **3**, Figure S4: Crystal structure of palladium by-product C_36_H_30_I_4_P_2_Pd_2_ (view along *c* axis) (grown by slow evaporation from hexane–ethylacetate solvent). C atoms are represented by gray spheres; I atoms, magenta; P atoms, green; and Pd atoms, blue, Figure S5: Fragments of ^1^H NMR spectra (6.7–4.5 ppm) of reaction mixtures of propargyl ether **2** with benzoyl chloride under different reaction conditions from Table 1 (CDCl_3_, 400 MHz, 298 K). Numbers indicate the integral intensity values of corresponding resonances. Yellow background highlights the region with resonances of unreacted propargyl aryl ether **2**; green background, diyne **4**; orange background, furan **5**; and blue background, ynone **6**, Figure S6: ^1^H NMR spectrum of compound **5** (CDCl_3_, 400 MHz, 298 K), Figure S7: ^13^C NMR spectrum and DEPT-135 experiment of compound **5** (CDCl_3_, 100 MHz, 298 K), Figure S8: ESI mass spectrum (0.1% NH_4_OAc, MeOH) of furan **5**, Figure S9: Fragment of ^1^H NMR spectrum (6.7–4.7 ppm) of reaction mixture of propargyl ether **2** with benzoyl chloride and hydrazine (CDCl_3_, 400 MHz, 298 K). Numbers indicate the integral intensity values of corresponding resonances of furan **5** at 6.64 and 5.67 ppm, pyrazole **7** at 5.13 ppm, and diyne **4** at 4.73 ppm, Figure S10: IR absorbance spectra of pyrazole **7** fractions in KBr pellet, Figure S11: ESI mass spectrum (0.1% NH_4_OAc, MeOH) of pyrazole **7”**, Figure S12: ^1^H NMR spectrum of compound **7** (fraction **7’**) (CDCl_3_, 600 MHz, 298 K), Figure S13: ^13^C NMR spectrum and DEPT-135 experiment of compound **7** (fraction **7’**) (CDCl_3_, 151 MHz, 298 K), Figure S14: ^1^H NMR spectrum of compound **7** (fraction **7”**) (CDCl_3_, 600 MHz, 298 K), Figure S15: ^13^C NMR spectrum and DEPT-135 experiment of compound **7** (fraction **7”**) (CDCl_3_, 151 MHz, 298 K), Table S1: Calculated total electronic energies, enthalpies, entropies, and Gibbs free energies (in Hartree) for optimized equilibrium model structures (*E*, *H*, *S*, and *G*, respectively).

## Sample Availability

Samples of compounds **3**, **5**, and **7** are available from the authors upon reasonable request.
