# Supplementary material for: Thermodynamic vs. Kinetic Control in Synthesis of O-Donor 2,5-Substituted Furan and 3,5-Substituted Pyrazole from Heteropropargyl Precursor
Source: Molecules. 2022 Aug 14;27(16):5178. doi: 10.3390/molecules27165178 (PMC9413326; doi:10.3390/molecules27165178)
Supplement: Supplementary file 1 [file molecules-27-05178-s001.zip › molecules-1849312-supplementary.pdf]

Thermodynamic vs. Kinetic Control in Synthesis of *O*-Donor 2,5-Substituted Furan and 3,5-Substituted Pyrazole from Heteropropargyl Precursor

Anton A. Muravev [1,2,*], Alexander S. Ovsyannikov [2], Gennady V. Konorov [3], Daut R. Islamov [2], Konstantin S. Usachev [4], Alexander S. Novikov [5,6], Svetlana E. Solovieva [2] and Igor S. Antipin [2]

[1] Infochemistry Scientific Center, ITMO University, 191002 St.-Petersburg, Russia
[2] Arbuzov Institute of Organic and Physical Chemistry, FRC Kazan Scientific Center, Russian Academy of Sciences, 420008 Kazan, Russia
[3] Butlerov Institute of Chemistry, Kazan Federal University, 420008 Kazan, Russia
[4] Institute of Fundamental Medicine and Biology, Kazan Federal University, 420008 Kazan, Russia
[5] Institute of Chemistry, St.-Petersburg State University, 199034 St.-Petersburg, Russia
[6] Joint Research Institute of Chemistry, Faculty of Physics, Mathematics and Natural Sciences, Peoples' Friendship University of Russia (RUDN University), 117198 Moscow, Russia
* Correspondence: muravev@itmo.ru

Electronic Supplementary Information

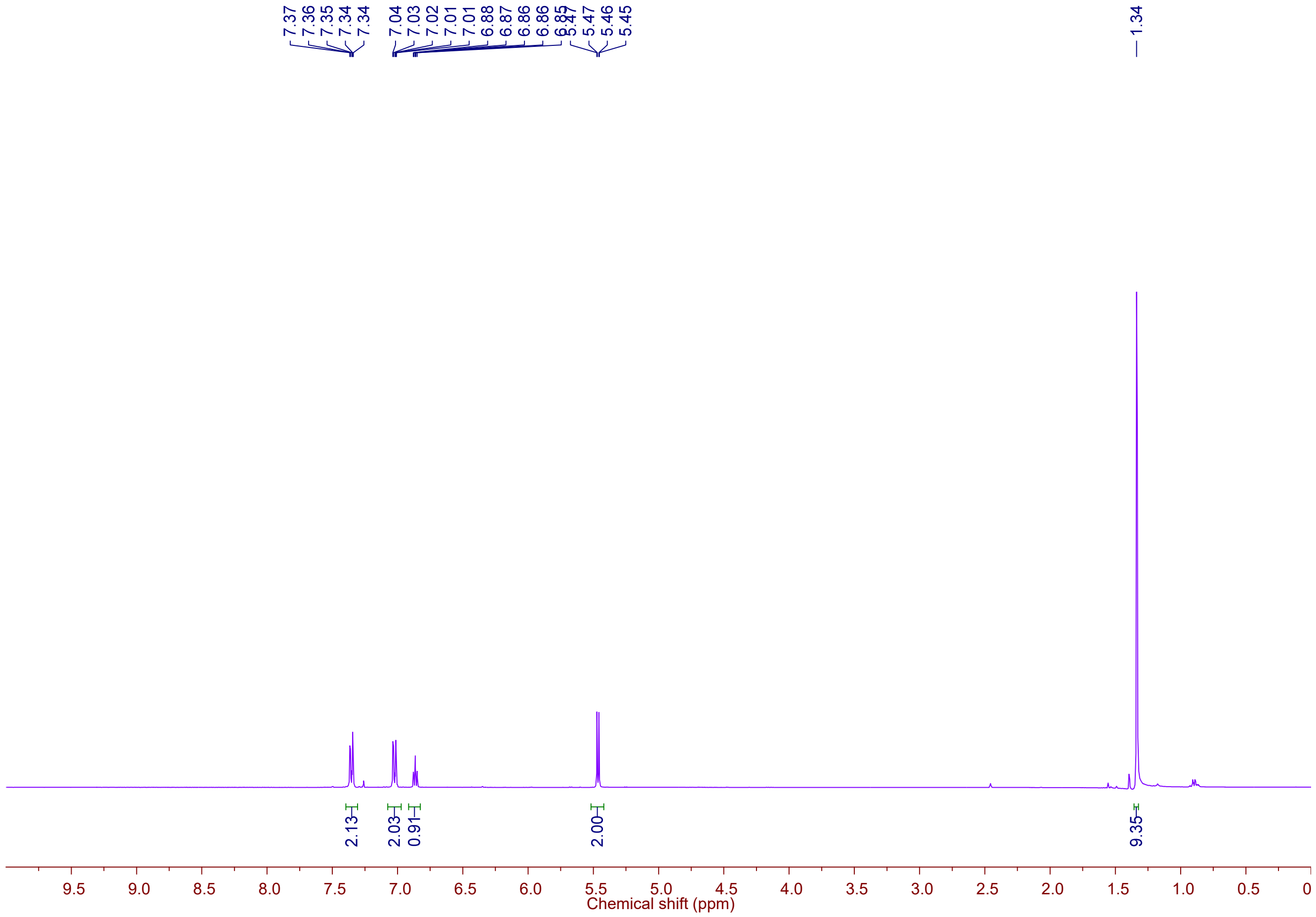

Figure S1. ^{1}H NMR spectrum of compound **3** ($CDCl_3$, 400 MHz, 298 K).

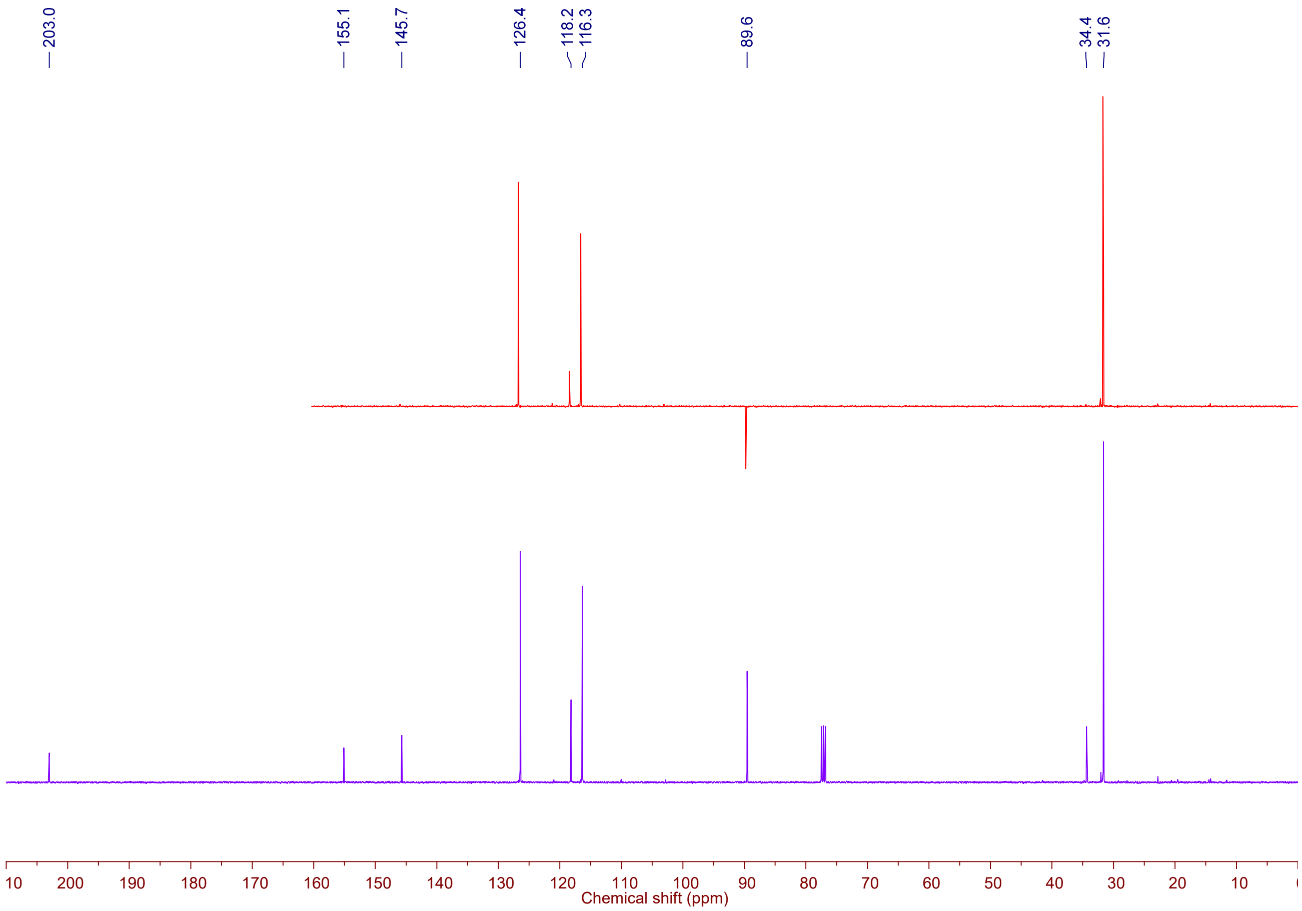

Figure S2. ^{13}C NMR spectrum and DEPT-135 experiment of compound **3** ($CDCl_3$, 100 MHz, 303 K).

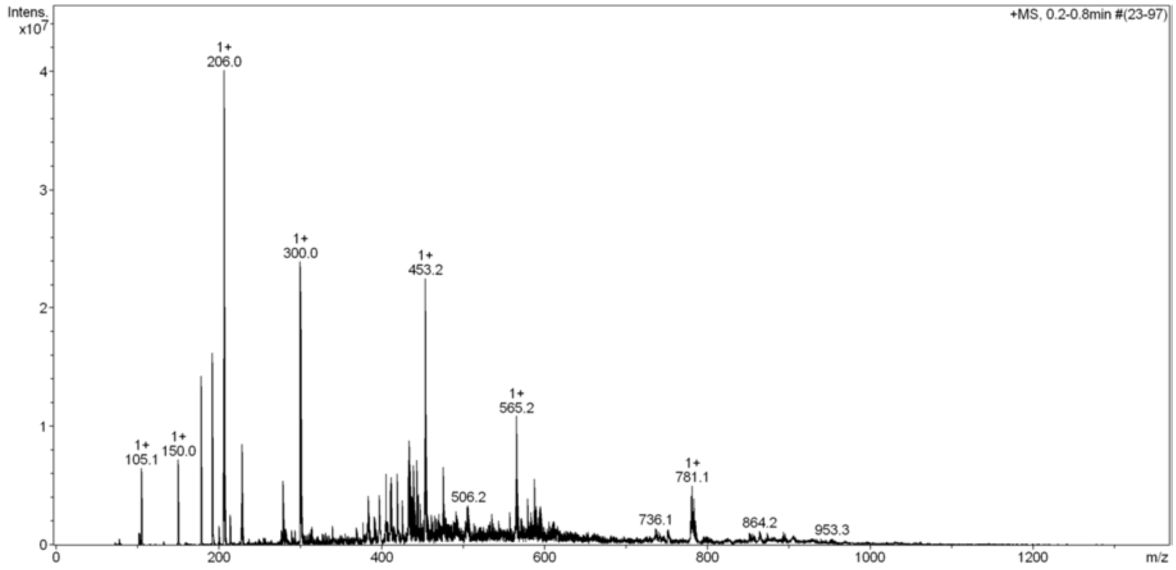

Figure S3. ESI mass spectrum (0.1% NH_4OAc, MeOH) of allene **3**.

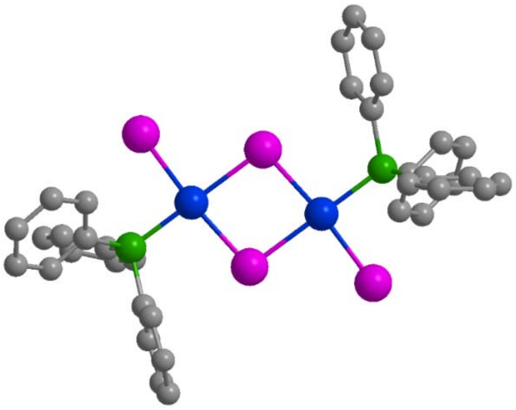

Figure S4. Crystal structure of palladium by-product $C_{36}H_{30}I_4P_2Pd_2$ (view along *c* axis) (grown by slow evaporation from hexane–ethylacetate solvent). C atoms are represented by gray spheres; I atoms, magenta; P atoms, green; and Pd atoms, blue.

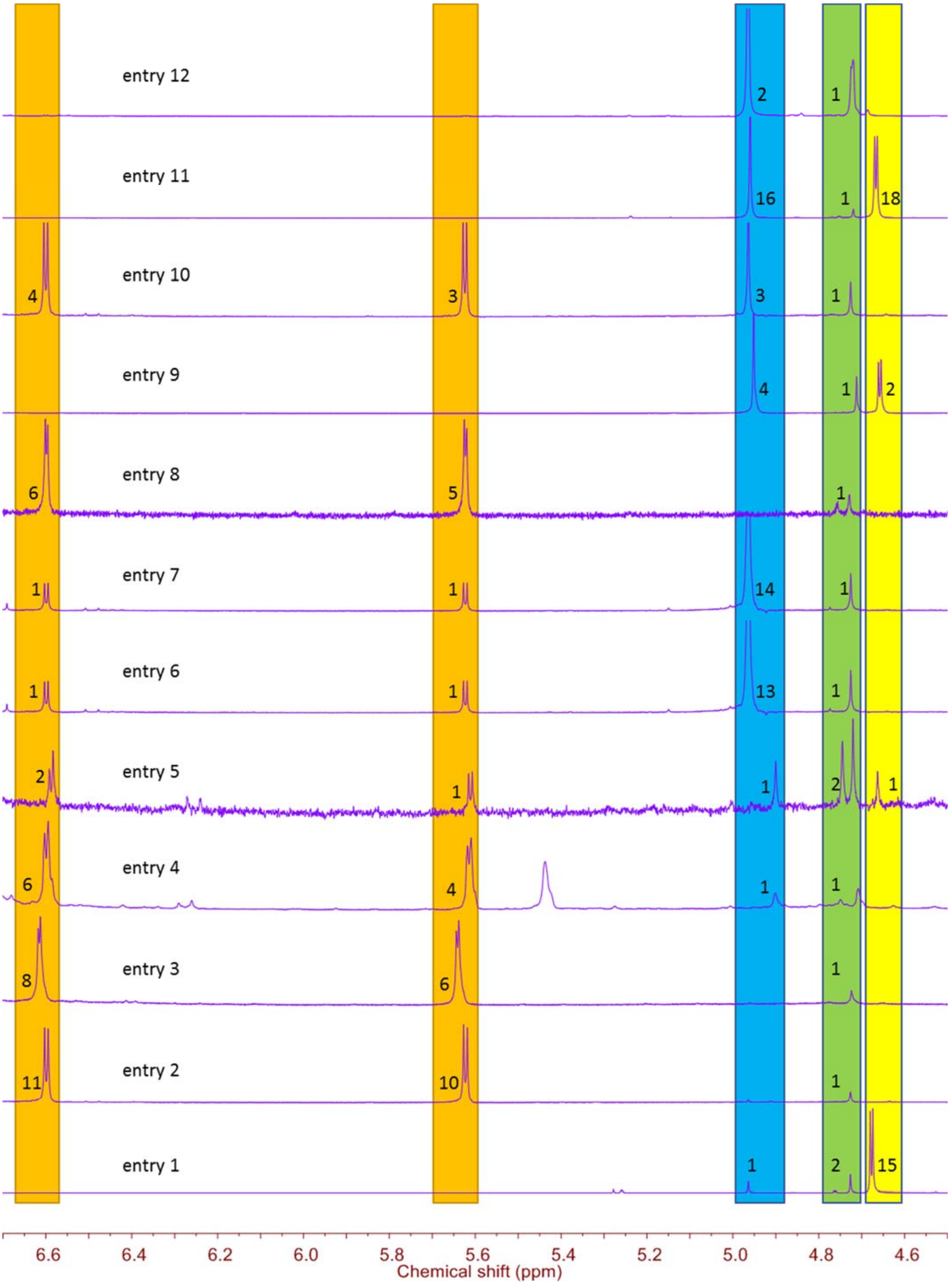

Figure S5. Fragments of ^{1}H NMR spectra (6.7–4.5 ppm) of reaction mixtures of propargyl ether **2** with benzoyl chloride under different reaction conditions from Table 1 ($CDCl_3$, 400 MHz, 298 K). Numbers indicate the integral intensity values of corresponding resonances. Yellow background highlights the region with resonances of unreacted propargyl aryl ether **2**; green background, diyne **4**; orange background, furan **5**; and blue background, ynone **6**.

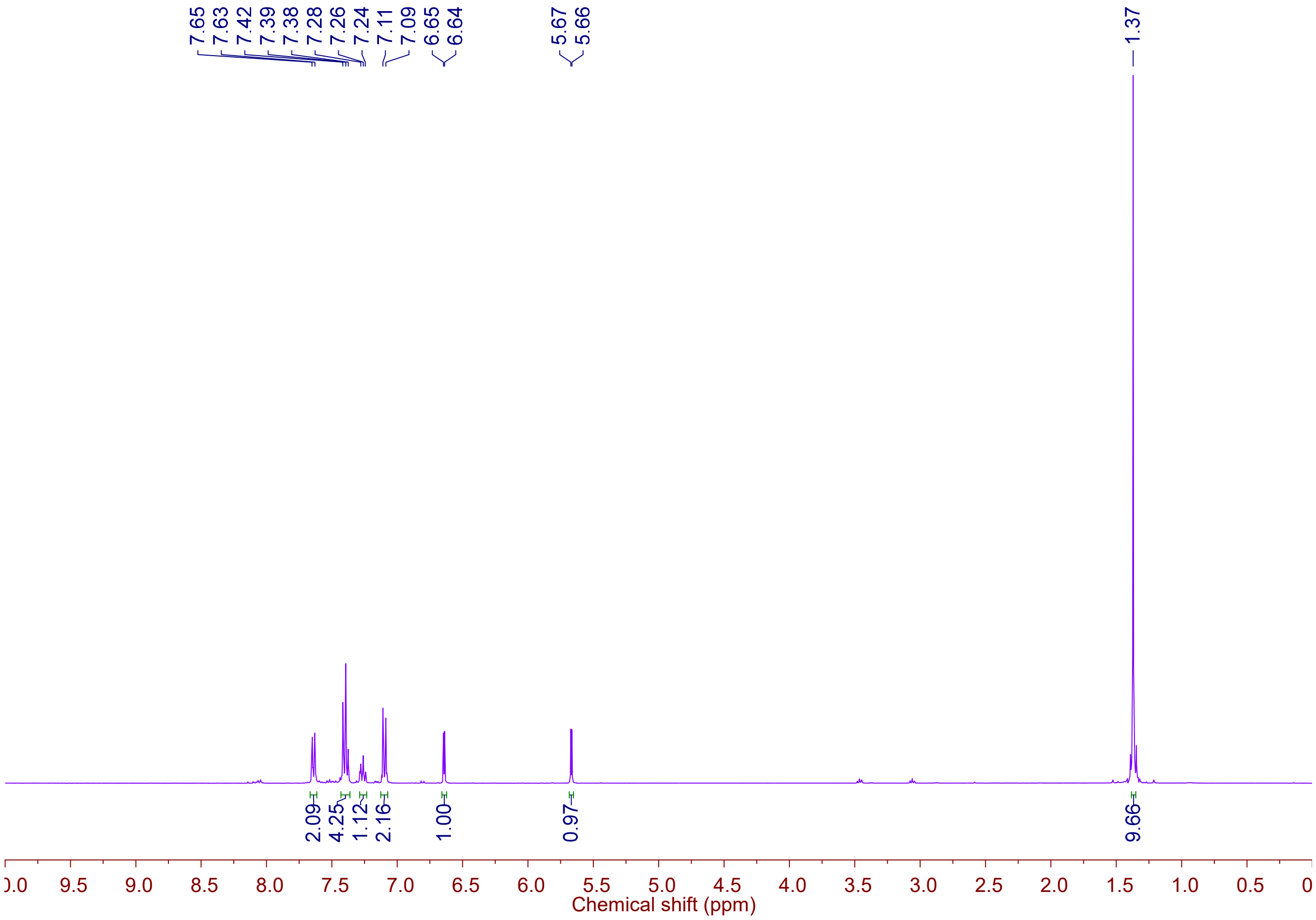

Figure S6. ^{1}H NMR spectrum of compound **5** ($CDCl_3$, 400 MHz, 298 K).

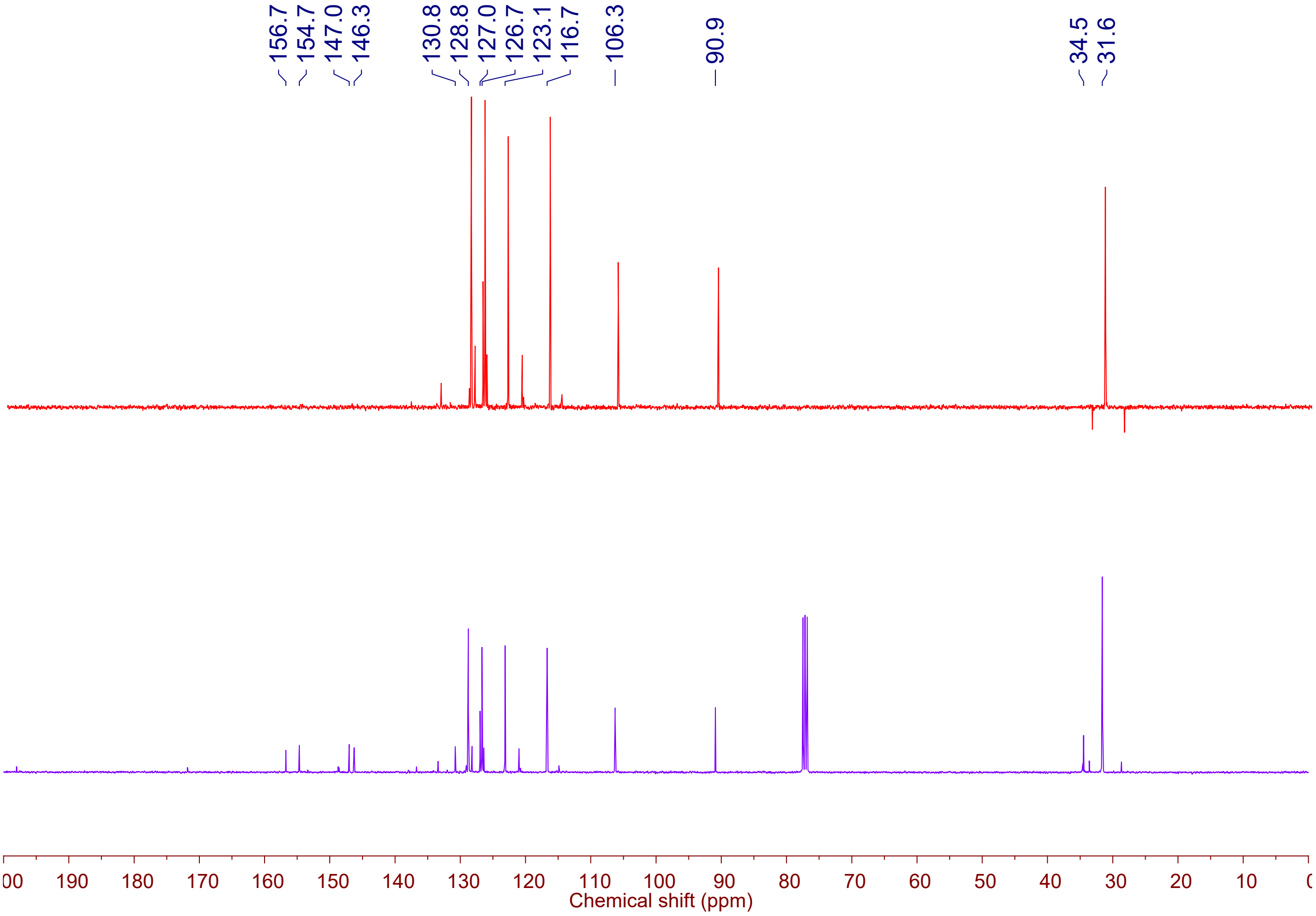

Figure S7. ^{13}C NMR spectrum and DEPT-135 experiment of compound **5** ($CDCl_3$, 100 MHz, 298 K).

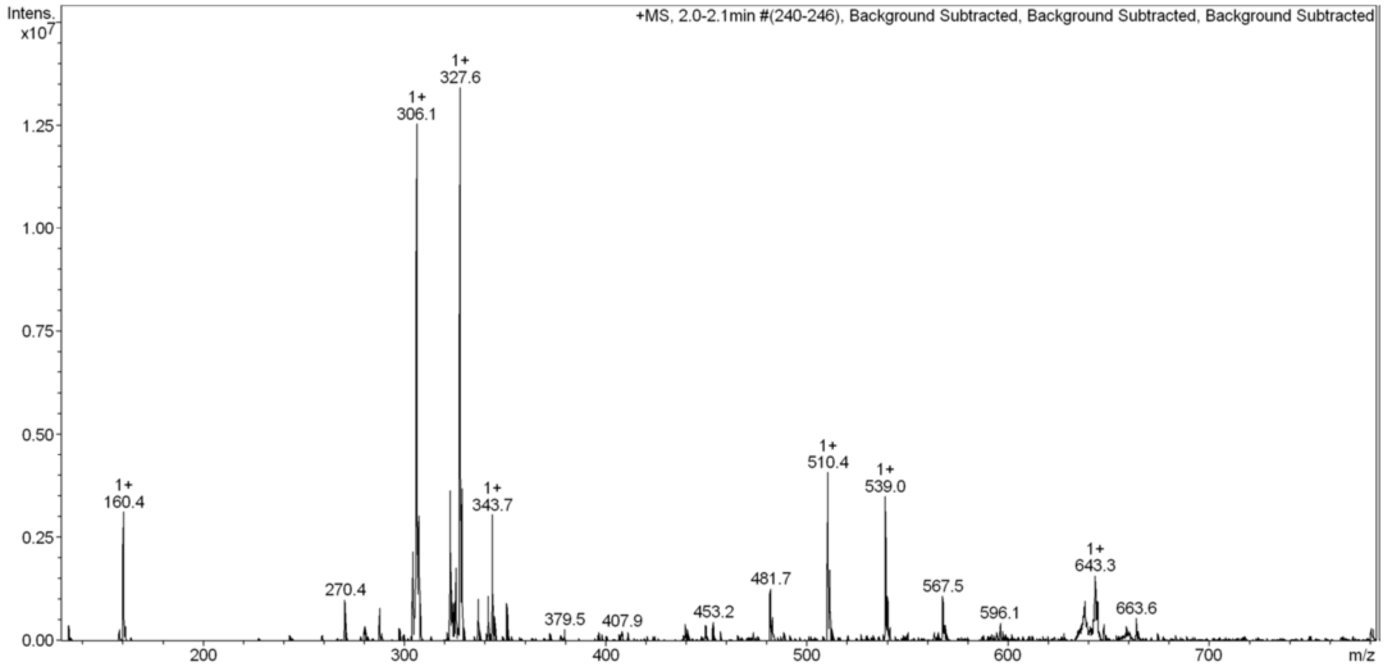

Figure S8. ESI mass spectrum (0.1% NH_4OAc, MeOH) of furan **5**.

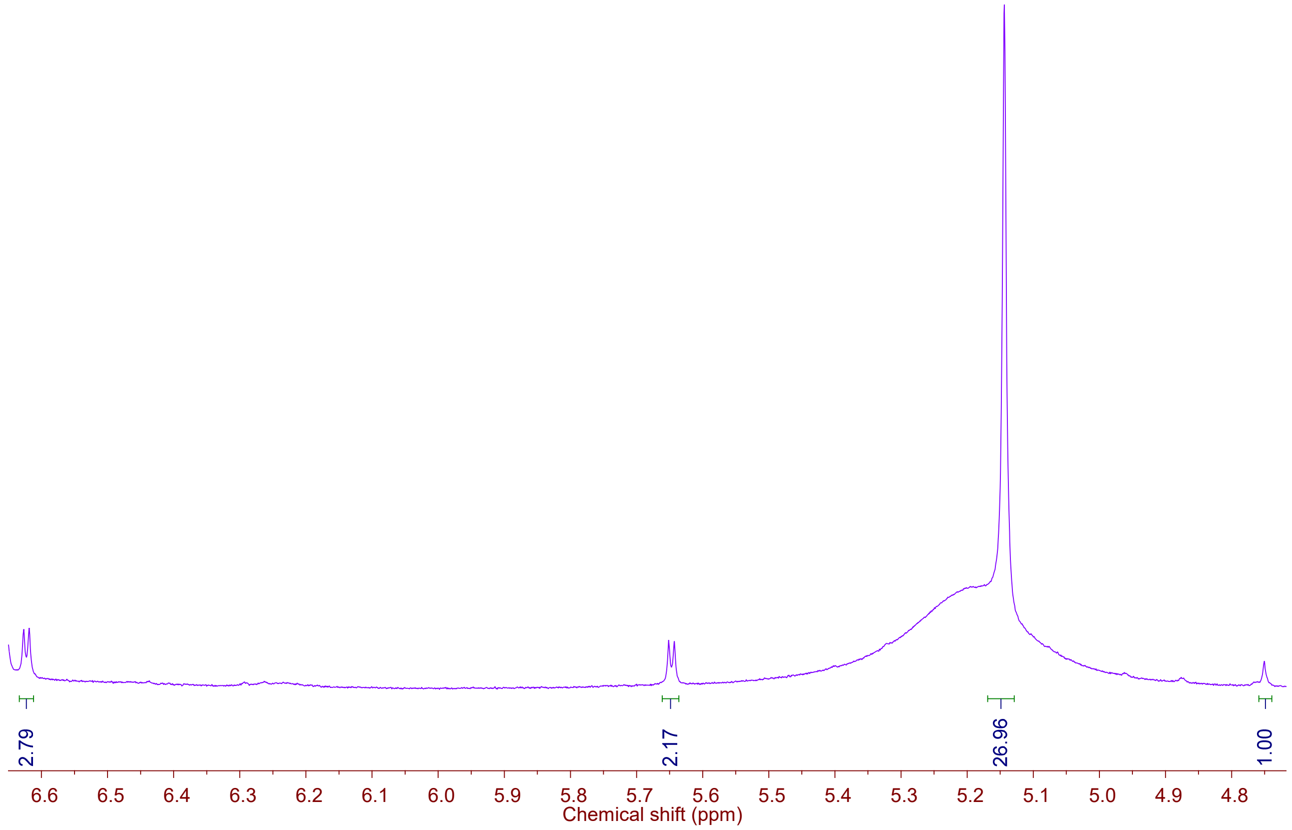

Figure S9. Fragment of ^{1}H NMR spectrum (6.7–4.7 ppm) of reaction mixture of propargyl ether **2** with benzoyl chloride and hydrazine ($CDCl_3$, 400 MHz, 298 K). Numbers indicate the integral intensity values of corresponding resonances of furan **5** at 6.64 and 5.67 ppm, pyrazole **7** at 5.13 ppm, and diyne **4** at 4.73 ppm.

Table S1. Calculated total electronic energies, enthalpies, entropies, and Gibbs free energies (in Hartree) for optimized equilibrium model structures (*E*, *H*, *S*, and *G*, respectively).

Model structure	*E*	*H*	*G*	*S*
1*H*-**7**	-959.680013901	-959.274348	-959.349233	157.610
2*H*-**7**	-959.678737122	-959.273387	-959.346978	154.886
MeOH	-115.654766919	-115.598249	-115.625091	56.493
$\mathbf{TS}_{inter\text{-}2}$	-1191.00758540	-1190.495006	-1190.582235	183.588
$\mathbf{TS}_{inter\text{-}1}$	-1075.30939661	-1074.851623	-1074.934614	174.670
$\mathbf{TS}_{intra}$	-959.597098951	-959.197804	-959.271315	154.715

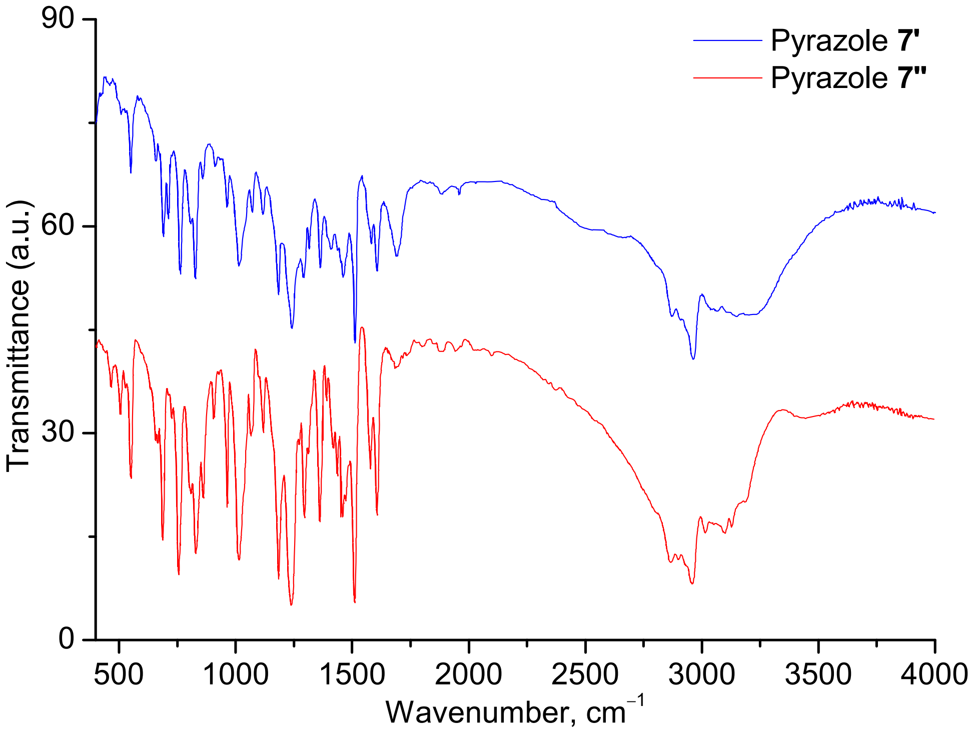

Figure S10. IR absorbance spectra of pyrazole **7** fractions in KBr pellet.

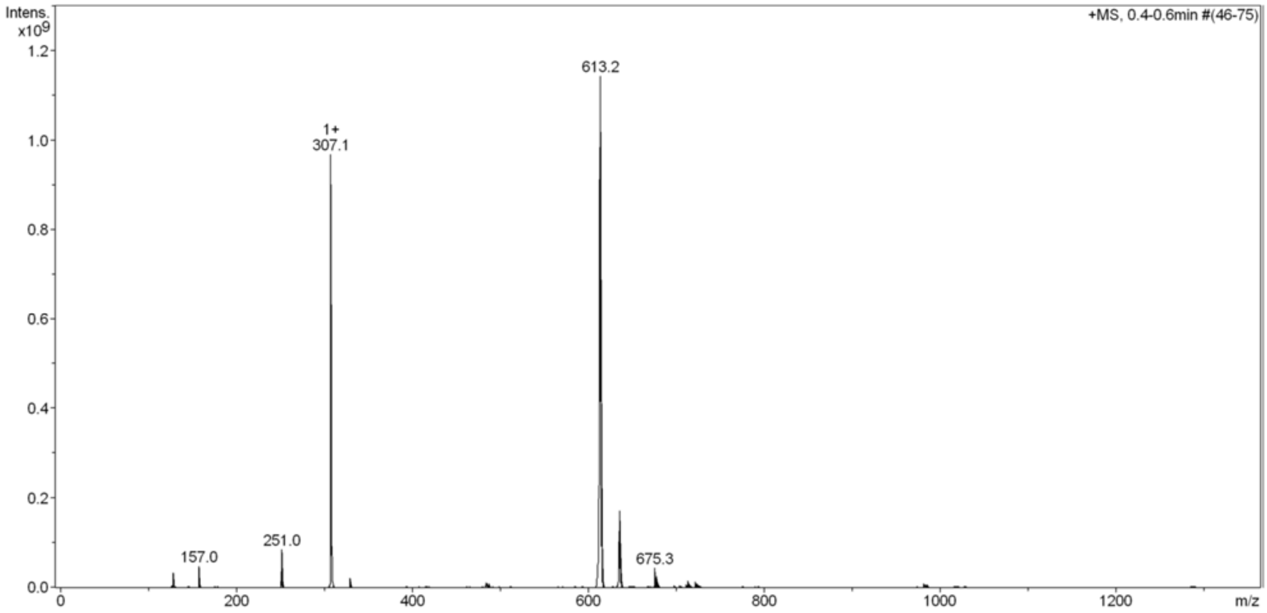

Figure S11. ESI mass spectrum (0.1% NH_4OAc, MeOH) of pyrazole **7”**.

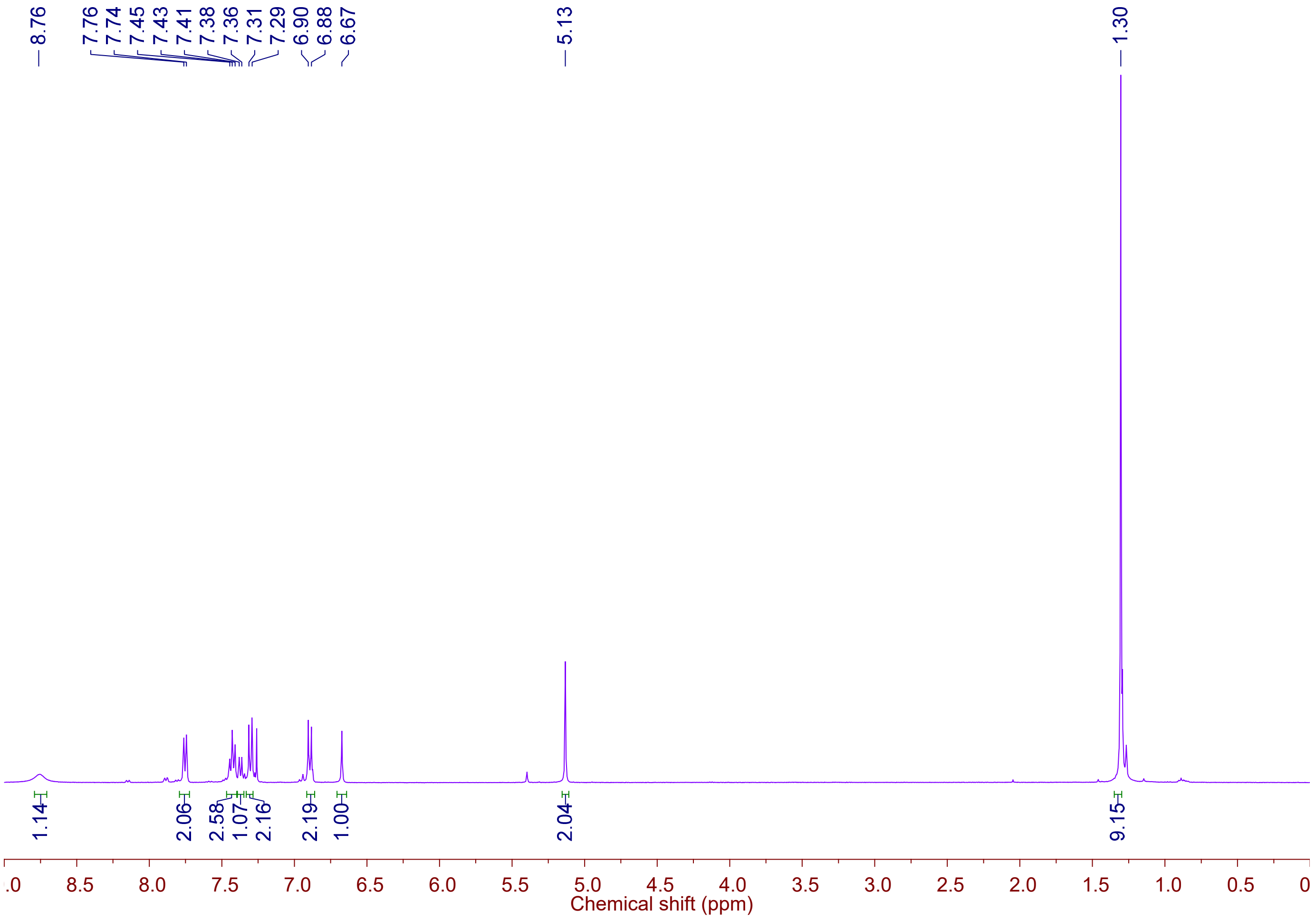

Figure S12. ^{1}H NMR spectrum of compound **7** (fraction **7’**) ($CDCl_3$, 600 MHz, 298 K).

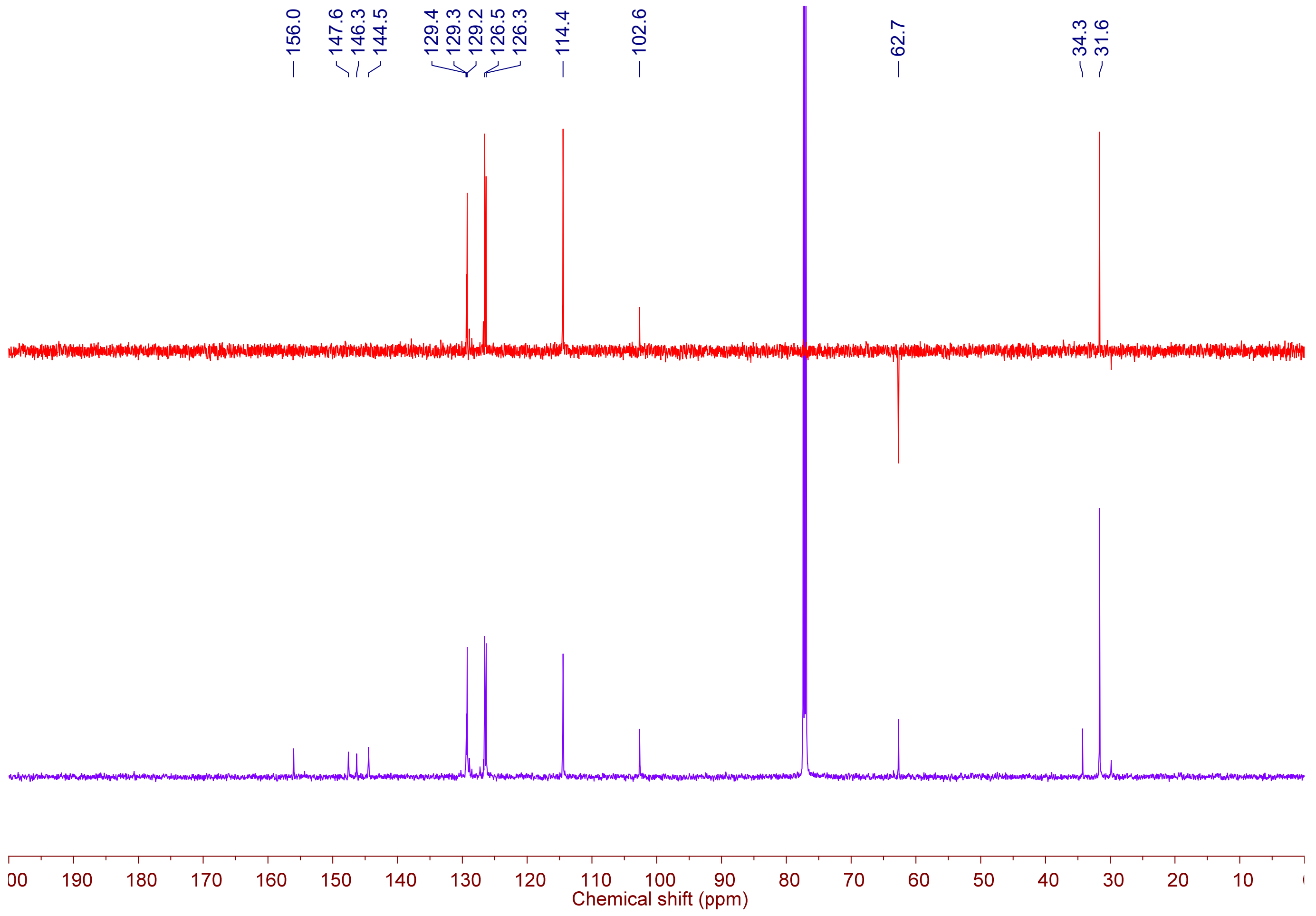

Figure S13. ^{13}C NMR spectrum and DEPT-135 experiment of compound **7** (fraction **7’**) ($CDCl_3$, 151 MHz, 298 K).

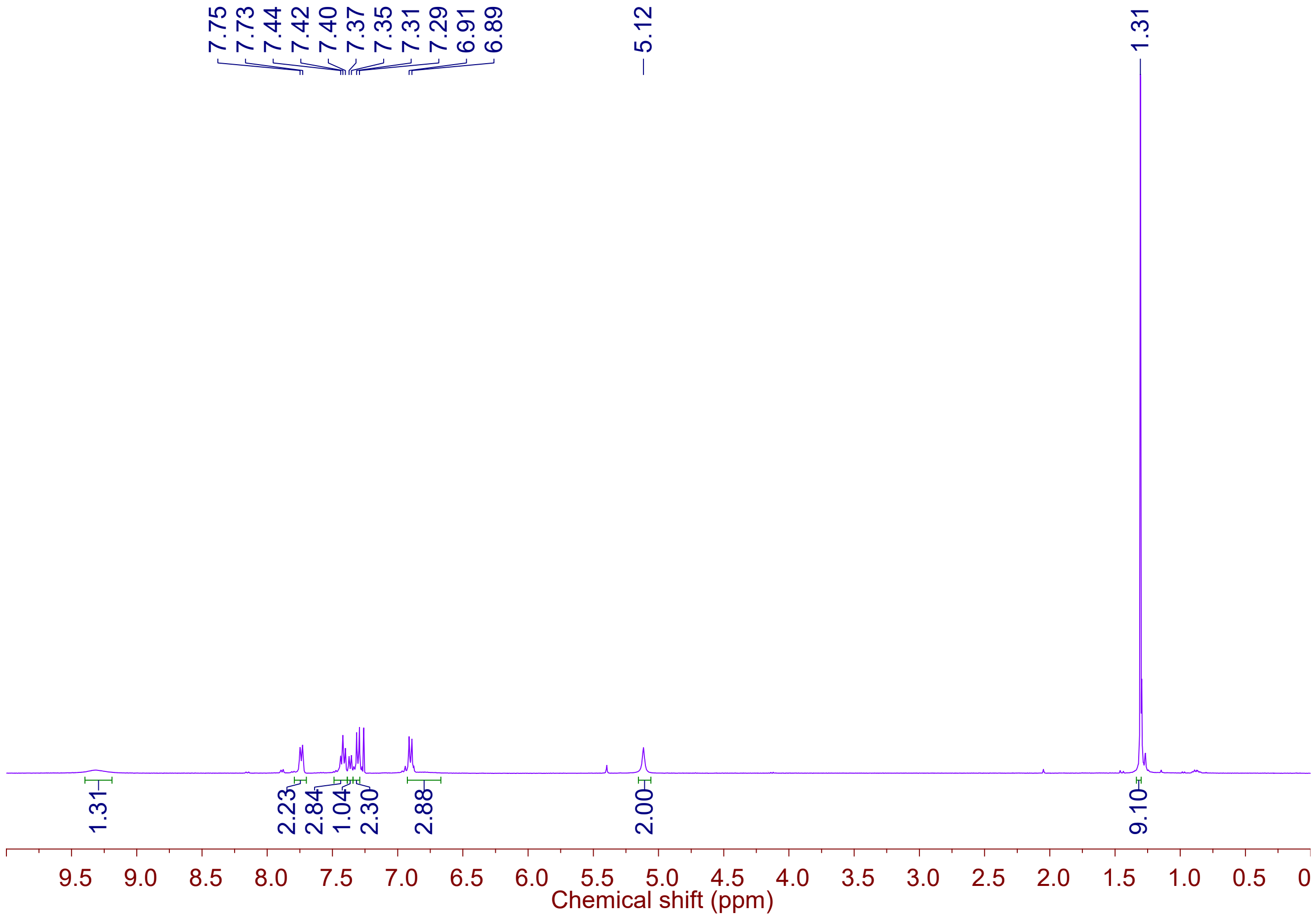

Figure S14. ^{1}H NMR spectrum of compound **7** (fraction **7”**) ($CDCl_3$, 600 MHz, 298 K).

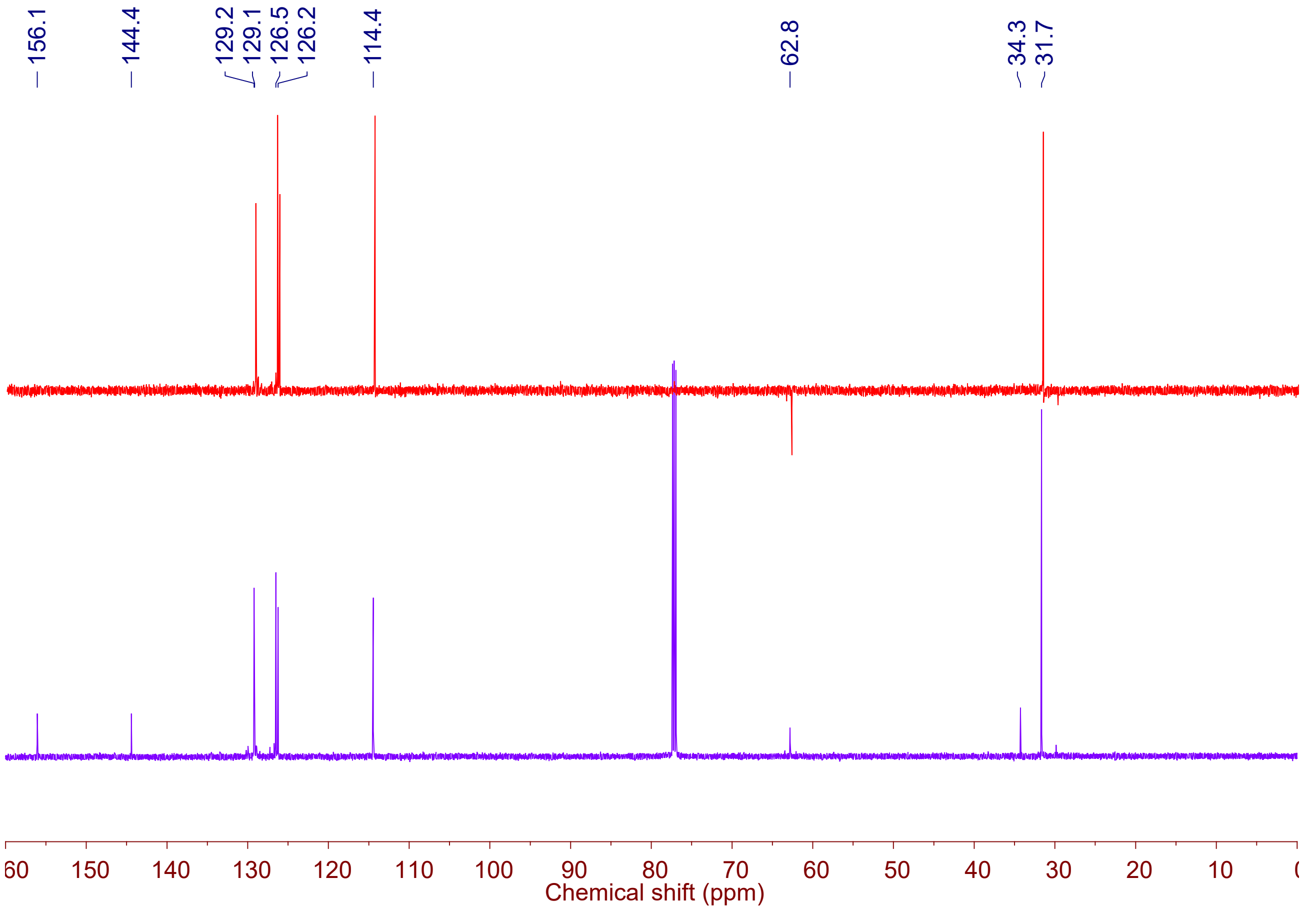

Figure S15. ^{13}C NMR spectrum and DEPT-135 experiment of compound **7** (fraction **7”**) ($CDCl_3$, 151 MHz, 298 K).
